# Adverse outcomes in patients with atrial fibrillation and a pacemaker: a cohort study

**DOI:** 10.1093/europace/euaf275

**Published:** 2025-10-28

**Authors:** Vincent Meier, Stefanie Aeschbacher, Michael Coslovsky, Andreas Gasser, Rebecca E Paladini, Tobias Reichlin, Nicolas Rodondi, Andreas Müller, Jürg Beer, Giulio Conte, Giorgio Moschovitis, Julia Bardoczi, Alain Bernheim, Elia Rigamonti, Laura Möri, Christine S Zuern, Felix Mahfoud, Christian Sticherling, David Conen, Stefan Osswald, Michael Kühne, Philipp Krisai

**Affiliations:** Department of Cardiology, University Hospital Basel, Petersgraben 4, Basel 4031, Switzerland; Cardiovascular Research Institute Basel, University Hospital Basel, Spitalstrasse 2, Basel 4056, Switzerland; Department of Cardiology, University Hospital Basel, Petersgraben 4, Basel 4031, Switzerland; Cardiovascular Research Institute Basel, University Hospital Basel, Spitalstrasse 2, Basel 4056, Switzerland; Department of Cardiology, University Hospital Basel, Petersgraben 4, Basel 4031, Switzerland; Department of Clinical Research, University of Basel, University Hospital Basel, Basel, Switzerland; Department of Cardiology, University Hospital Basel, Petersgraben 4, Basel 4031, Switzerland; Cardiovascular Research Institute Basel, University Hospital Basel, Spitalstrasse 2, Basel 4056, Switzerland; Department of Cardiology, University Hospital Basel, Petersgraben 4, Basel 4031, Switzerland; Cardiovascular Research Institute Basel, University Hospital Basel, Spitalstrasse 2, Basel 4056, Switzerland; Department of Cardiology Inselspital, Bern University Hospital, University of Bern, Bern, Switzerland; Department of General Internal Medicine Inselspital, Bern University Hospital, University of Bern, Bern, Switzerland; Institute of Primary Health Care (BIHAM), University of Bern, Bern, Switzerland; Department of Cardiology, Triemli Hospital Zürich, Zürich, Switzerland; Department of Medicine, Cantonal Hospital of Baden and Molecular Cardiology, University Hospital of Zürich, Zürich, Switzerland; Cardiocentro Ticino Institute, Ente Ospedaliero Cantonale Lugano (EOC), Lugano, Switzerland; Cardiocentro Ticino Institute, Ente Ospedaliero Cantonale Lugano (EOC), Lugano, Switzerland; Department of General Internal Medicine Inselspital, Bern University Hospital, University of Bern, Bern, Switzerland; Institute of Primary Health Care (BIHAM), University of Bern, Bern, Switzerland; Department of Cardiology, Triemli Hospital Zürich, Zürich, Switzerland; Cardiocentro Ticino Institute, Ente Ospedaliero Cantonale Lugano (EOC), Lugano, Switzerland; Department of Cardiology, University Hospital Basel, Petersgraben 4, Basel 4031, Switzerland; Cardiovascular Research Institute Basel, University Hospital Basel, Spitalstrasse 2, Basel 4056, Switzerland; Department of Cardiology, University Hospital Basel, Petersgraben 4, Basel 4031, Switzerland; Cardiovascular Research Institute Basel, University Hospital Basel, Spitalstrasse 2, Basel 4056, Switzerland; Department of Cardiology, University Hospital Basel, Petersgraben 4, Basel 4031, Switzerland; Cardiovascular Research Institute Basel, University Hospital Basel, Spitalstrasse 2, Basel 4056, Switzerland; Department of Cardiology, University Hospital Basel, Petersgraben 4, Basel 4031, Switzerland; Cardiovascular Research Institute Basel, University Hospital Basel, Spitalstrasse 2, Basel 4056, Switzerland; Population Health Research Institute, McMaster University, Hamilton, ON, Canada; Department of Cardiology, University Hospital Basel, Petersgraben 4, Basel 4031, Switzerland; Cardiovascular Research Institute Basel, University Hospital Basel, Spitalstrasse 2, Basel 4056, Switzerland; Department of Cardiology, University Hospital Basel, Petersgraben 4, Basel 4031, Switzerland; Cardiovascular Research Institute Basel, University Hospital Basel, Spitalstrasse 2, Basel 4056, Switzerland; Department of Cardiology, University Hospital Basel, Petersgraben 4, Basel 4031, Switzerland; Cardiovascular Research Institute Basel, University Hospital Basel, Spitalstrasse 2, Basel 4056, Switzerland

**Keywords:** Atrial fibrillation, Pacemaker, MACE, Adverse outcomes

## Abstract

**Aims:**

Patients with atrial fibrillation (AF) are at a high risk of adverse cardiovascular outcomes. Little is known about the specific population of AF patients with implanted pacemaker (PM) and their prognosis. Therefore, we aimed to compare the risks of adverse outcomes in AF patients with and without PM.

**Methods and results:**

Data from two Swiss prospective, multicentre cohort studies (Swiss-AF, Beat-AF) (*n* = 3675) with yearly follow-ups (FUs) up to 8 years were analysed. The first main outcome was major adverse cardiovascular events (MACE), a composite of stroke or transient ischaemic attack, myocardial infarction, cardiovascular death, and systemic embolism. The second main outcome was hospitalization for heart failure (HF). Secondary outcomes were the individual components of MACE. We performed time-updated Cox regression analyses to investigate the association of PM and outcomes. Median age was 71.4 years, 28.8% female, 445 (12.1%) patients had a PM at baseline, and 238 additional patients (7.4%, 1.05%/year) received a PM over a median FU of 7 years. Patients with a PM had higher incidence rates for MACE and HF (5.97 and 5.08 per 100 patient-years, respectively), compared to patients without a PM (3.37 and 2.61 per 100 patient-years, respectively). After multivariable adjustment, we found no independent association of PM and MACE (aHR [95% CI] 1.12 [0.95–1.33; *P* = 0.183]) or HF (aHR [95% CI] 1.14 [0.94–1.37; *P* = 0.180]). We found consistent results for the individual components of MACE.

**Conclusion:**

Patients with AF and a PM experienced an increased rate of adverse cardiovascular outcomes. However, the PM itself was not independently associated with these outcomes.

## Graphical Abstract

**Graphical Abstract:**
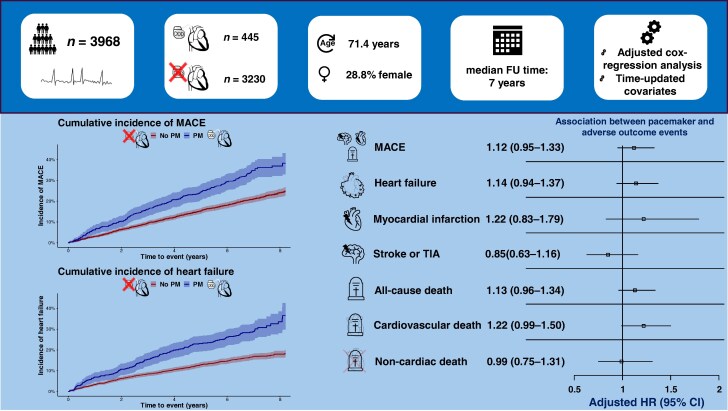

What’s new?

- This is the first study examining the associations of pacemakers (PMs) to adverse outcomes in patients with atrial fibrillation (AF)
- We show that patients with AF and a PM had substantially higher event rates for major adverse cardiovascular events and hospitalization for heart failure (HF) compared to AF patients without PM
- After adjustment, the presence of PM itself was not independently associated with adverse outcomes
- The data suggests that AF patients with a PM represent a high-risk group within the AF population and may therefore benefit from more intensive follow-up and target treatment strategies to improve their clinical outcomes

## Introduction

Patients with atrial fibrillation (AF) are at an increased risk of sinus node dysfunction^1–3^ and are more likely to experience atrioventricular (AV) block.^4^ Consequently, the rate of pacemaker (PM) implantation is higher in patients with AF compared to those without AF.^5–7^ Moreover, beyond intrinsic mechanisms, some AF patients may undergo PM implantation before AV node ablation as part of a pace-and-ablate strategy for rate control.^8,9^

As AF is the most common sustained arrhythmia, with its prevalence expected to rise in the coming decades, clinicians will increasingly encounter a substantial patient population with both AF and a PM.^10–12^ While several studies have investigated the prognostic implications of incident AF in patients with PMs, to the best of our knowledge, no prior study has examined the associations between the presence of a PM and adverse cardiovascular outcomes in patients with prevalent AF.^13–16^

The presence of a PM in AF patients may identify patients at differing risks for adverse cardiovascular outcomes compared to AF patients without a PM. This distinction may arise from underlying cardiac conditions affecting the conduction system, such as atrial cardiomyopathy with fibrosis of the conduction system, which consequently can lead to a PM implantation^17^ and may be associated with worse cardiovascular outcomes. Conversely, a PM might attenuate the risk of heart failure (HF) by ensuring a regular heart rhythm after AV node ablation^18^ or by maintaining an adequate minimum heart rate in patients with bradyarrhythmias but may also potentially worsen HF by inducing ventricular desynchrony.^7,19,20^ Given that AF patients already face an increased risk for various cardiovascular outcomes compared to the general population, any increase or attenuation of this risk may be clinically relevant.^21–23^

This study aimed at investigating the associations between the presence of a PM and the risks of major adverse cardiovascular events (MACE) and HF hospitalizations in a large, unselected cohort of patients with AF.

## Methods

### Study design and patient population

For this analysis, data from the Basel Atrial Fibrillation (Beat-AF) and the Swiss Atrial Fibrillation (Swiss-AF; NCT02105844) cohort studies were combined. Both cohorts are ongoing large prospective, observational, multicentre studies in Switzerland. The detailed methodologies have been published previously and were similar between the two studies.^24,25^ In Beat-AF, 1553 AF patients were enrolled between 2010 and 2014 across seven centres, while Swiss-AF enrolled 2415 AF patients from 2014 to 2017 across 14 centres. The main inclusion criterion for both cohorts was established AF. In Swiss-AF, an age ≥65 years was mandatory (except for a limited number of patients aged 45–65 years to study health economic aspects), whereas no age criterion was used in Beat-AF. Main exclusion criteria were the inability to sign informed consent, short reversible forms of AF (due to secondary causes such as infection or surgery), or any acute illness in the last 4 weeks. Patients with acute illness were eligible for enrolment after stabilization or resolution of the underlying condition. Patients enrolled in Beat-AF were not eligible to participate in Swiss-AF. The study was approved by local ethics committees in the participating centres (lead ethics committee: Ethikkommission Nordwest- und Zentralschweiz) and complied with the Helsinki declaration. Informed written consent was obtained from each participant. The data underlying this article will be shared on reasonable request to the corresponding author.

**Figure 1:**
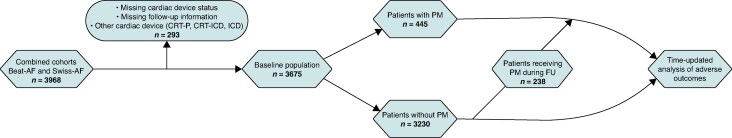
Study enrolment flow.

From a total of 3968 patients, we excluded 293 patients: 1 for missing information on cardiac device status, 67 due to missing follow-up (FU) information, and 218 due to having another cardiac device than a PM [e.g. cardiac resynchronization therapy (CRT), CRT with defibrillator (CRT-ICD), or implantable cardioverter-defibrillator (ICD)]. Patients with other cardiac devices than PM were excluded due to assumed differences in the underlying cardiac disease. Patients receiving another device than PM during the observational period were censored from the analysis at time of receiving the different device (*n* = 14) to avoid potential bias.^26^ Patients with implanted loop recorders (*n* = 24) were included in the group without PM. This resulted in 3675 patients included in this analysis. Patients receiving a PM implantation during the observation period crossed over into the PM group at this moment in time. Any crossovers were accounted for by the time-updated analysis. The study population enrolment flowchart is presented in *Figure 1*.

### Data collection

In both cohorts, information on individual patient characteristics, medical history including comorbidities, cardiovascular risk factors, lifestyle factors, current medication, and cardiac device implantations were collected using standardized case report forms. The CHA_2_DS_2_-VASc score was calculated [congestive HF (1 point), hypertension (1 point), age ≥ 75 years (2 points), diabetes (1 point), prior stroke/transient ischaemic attack/thromboembolism (2 points), vascular disease (1 point), age 65–74 years (1 point), and female sex (1 point)]. Smoking status was categorized as active vs non-active smokers. Body mass index (BMI) was calculated as weight in kilograms divided by height in metres squared. Blood pressure was measured three times in a supine position and the mean of all values was used. Atrial fibrillation type was categorized as paroxysmal, persistent, and permanent according to the AF guidelines at the time of study inception. In this analysis, paroxysmal and persistent AF were grouped together as non-permanent.^27^ The collected data were updated during yearly FU visits either in person or by phone.

### Assessment of pacemaker status

Information on device implantation was obtained at enrolment and in yearly FU visits. Details recorded were the type of the cardiac device, the date of implantation, and any complications related to device implantation. Cardiac device status was updated yearly up to 8 years after enrolment in Beat-AF and up to 7 years after enrolment in Swiss-AF.

### Outcome events

The two main outcome events were MACE and hospitalization for HF. Major adverse cardiovascular events included any stroke and/or transient ischaemic attack (TIA), systemic embolism (SE), myocardial infarction (MI), and cardiovascular death. Secondary outcomes were all-cause mortality, cardiovascular death, non-cardiovascular death, stroke, and/or TIA and MI. Outcomes were collected similarly in both cohorts. Detailed definitions of the main and secondary outcomes are provided in the supplement. All outcome events were independently validated by two trained physicians. In case of discrepancies, a third physician was consulted.

### Statistical analysis

Baseline characteristics were stratified by the presence or absence of a PM at baseline. Categorical variables were presented as numbers and percentages and compared using χ² test. Continuous variables were presented as mean ± standard deviation or median (interquartile range) and compared using a *t* test or the Mann–Whitney *U* test if strongly skewed. Patient-years (py) of FU were calculated as the difference between baseline and study termination (death, drop-out, or loss to FU), the respective outcome event or last visit date. Incidence rates were calculated as the number of events per 100 py of FU.

To investigate the associations of having a PM with adverse outcome events, we constructed time-updated Cox proportional hazards regression models to calculate hazard ratios (HRs) and 95% confidence intervals (CIs) and to adjust for potential confounders. A PM (yes/no) was used as the predictor of interest, and the variable status was updated whenever a participant received a PM device or if the participant changed from PM to another device. Model 1 was adjusted for age and sex. Model 2 was additionally adjusted for hypertension, diabetes, type of AF (permanent vs non-permanent), history of HF, history of stroke or TIA, history of MI, history of renal failure, BMI, smoking status (active vs non-smoking), oral anticoagulation, and antithrombotic treatment. In subgroup analyses, we repeated the analyses for the main outcomes stratified by AF type.

All analyses were performed using RStudio version 4.2.2.

### Patient and public involvement

Patients were asked at baseline if they wished to receive results of the studies and analyses conducted with the Beat-AF and Swiss-AF cohort. Brief result information will be sent by mail and e-mail or handed out during FU visits.

We used the Strengthening the Reporting of Observational Studies in Epidemiology (STROBE) cohort checklist when writing our report.^28^

## Results

Baseline characteristics, stratified by presence or absence of a PM at enrolment, are presented in *Table 1*. Overall, the mean age was 71.4 (±10) years and 1060 (28.8%) were female. Compared to patients without PM, patients with PM were older (77 vs 71 years), were more often female (32.8% vs 28.3%), and had more often diabetes (18.7% vs 14.9%), prior stroke or TIA (20.9% vs 16.8%), a history of HF (31.9% vs 19.5%), coronary artery disease (35.7% vs 23.5%), and chronic kidney disease (28.6% vs 15.7%). Patients with a history of PM implantation were more likely to receive oral anticoagulant (OAC) (88.8% vs 82.9%) and had a higher proportion of vitamin K antagonist (VKA) for anticoagulation (56.9% vs 46.4%). There was no difference in the prevalence of antiplatelet therapy.

**Table 1:** Baseline characteristics stratified by pacemaker status at study enrolment

Variable	Overall	No PM	PM	*P*-value
*n* (%)	3675	3230 (87.9)	445 (12.1)	–
Female sex (%)	1060 (28.8)	914 (28.3)	146 (32.8)	0.06
Age [mean (SD)]	71.4 (10.1)	70.6 (10.1)	77.1 (7.7)	<0.001
BMI [mean (SD)]	27.4 (4.7)	27.5 (4.8)	27.2 (4.3)	0.26
AF type (%)	–	–	–	<0.001
Permanent	815 (22.2)	667 (20.7)	148 (33.3)	–
Non-permanent	2858 (77.8)	2561 (79.3)	297 (66.7)	–
CHA_2_DS_2_-VASc Score [mean (SD)]	3.2 (1.8)	3.1 (1.8)	4.0 (1.5)	<0.001
Active smoking (%)	288 (7.8)	264 (8.2)	24 (5.4)	0.05
Hypertension (%)	2532 (68.9)	2196 (68.0)	336 (75.5)	0.002
Diabetes (%)	565 (15.4)	482 (14.9)	83 (18.7)	0.05
History of heart failure (%)	776 (21.1)	634 (19.6)	142 (31.9)	<0.001
History of stroke or TIA (%)	637 (17.3)	544 (16.8)	93 (20.9)	0.04
History of peripheral artery disease (%)	265 (7.2)	228 (7.1)	37 (8.3)	0.39
History of coronary heart disease (%)	919 (25.0)	760 (23.5)	159 (35.7)	<0.001
History of myocardial infarction (%)	482 (13.1)	394 (12.2)	88 (19.8)	<0.001
History of renal failure (%)	633 (17.2)	506 (15.7)	127 (28.6)	<0.001
Medication				
OAC (%)	3072 (83.6)	2677 (82.9)	395 (88.8)	0.002
VKA (%)	1750 (47.6)	1497 (46.4)	253 (56.9)	<0.001
DOAC (%)	1320 (35.9)	1178 (36.5)	142 (31.9)	0.07
Antiplatelet therapy (%)	773 (21.1)	670 (20.8)	103 (23.3)	0.27

Values are mean SD, standard deviation; AF, atrial fibrillation; BMI, body mass index; DOAC, direct oral anticoagulants; OAC, oral anticoagulant; PM, pacemaker; TIA, transient ischaemic attack; VKA, vitamin K antagonist.

### Main outcomes

**Figure 2:**
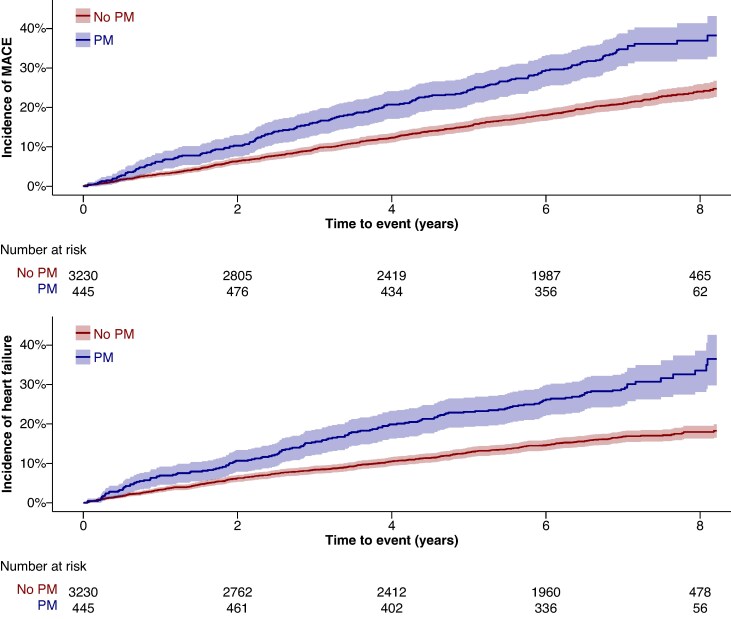
Cumulative incidence of MACE (left) and cumulative incidence of heart failure hospitalizations (right). MACE, major adverse cardiovascular events: composite of stroke and/or transient ischaemic attack, systemic embolism, myocardial infarction, cardiovascular mortality; PM, pacemaker.

**Figure 3:**
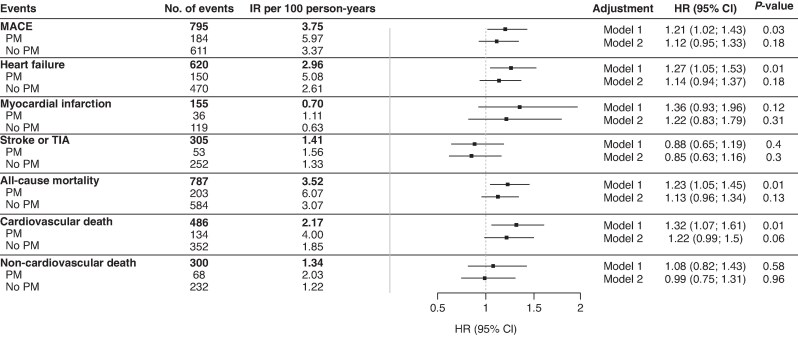
Association between pacemaker status and adverse outcome events. No., number; IR, incidence rate; MACE, major adverse cardiovascular events: composite of stroke and/or transient ischaemic attack, systemic embolism, myocardial infarction, cardiovascular mortality; HR (95% CI), hazard ratio (95% confidence interval) for MACE and heart failure in patients with pacemaker; PM, pacemaker; Model 1: adjusted for age and sex. Model 2: additionally adjusted for hypertension, diabetes, type of atrial fibrillation, history of heart failure, history of stroke or TIA, history of myocardial infarction, history of renal failure, BMI, active smoking, anticoagulation, and antithrombotic treatment.

Over a median FU time of 7.0 years (IQR, 6.0–7.9), an additional 238 patients (7.4%, 1.05%/year) received a PM and therefore crossed over into the PM group at the time of implantation. Overall, there were 795 (3.75 per 100 py) MACE outcomes, 184 in patients with PM and 611 in patients without PM, translating into incidence rates of 5.97 and 3.37 per 100 py, respectively. Cumulative incidence curves for MACE, stratified by PM status, are presented in *Figure 2*. In the time-updated Cox regression model, the multivariable-adjusted HR (95% CI) of the presence of a PM for MACE was 1.12 (0.95–1.33; *P* = 0.183) (*Figure 3*).

The second main outcome of HF hospitalizations occurred in 620 patients (2.96 per 100 py) overall: 150 in patients with PM and 470 in patients without PM, translating into incidence rates of 5.08 and 2.61 per 100 py, respectively (*Figure 2*). After multivariable adjustment, the HR (95% CI) for HF hospitalizations was 1.14 (0.94–1.37; *P* = 0.180) (*Figure 3*).

In subgroup analyses, there was no modifying effect of AF type for the associations of PM with MACE (*P* = 0.47) or HF (*P* = 0.95) (*Table 2*).

**Table 2:** Subgroup analysis of the modifying effect of atrial fibrillation type on the associations with pacemaker

Outcome	No. of events	Incidence rate per 100 person-years	AF type	PM	*P*-value
				HR (95% CI)	
MACE	318	6.24	Permanent	1.21 (0.94–1.56)	0.47
	477	2.96	Non-permanent	1.04 (0.83–1.31)	
Heart failure	244	4.97	Permanent	0.90 (0.65–1.23)	0.95
	376	2.35	Non-permanent	1.30 (1.03–1.65)	

AF, atrial fibrillation; MACE, major adverse cardiovascular events: composite of stroke and/or transient ischaemic attack, systemic embolism, myocardial infarction, cardiovascular mortality; HR (95% CI), hazard ratio (95% confidence interval) for MACE and heart failure in patients with pacemaker; PM, pacemaker.

### Secondary outcomes

Incidence rates and regression models for the secondary outcomes are shown in *Figure 3*. All secondary outcomes occurred more often in patients with a PM compared to patients without a PM: 1.11 vs 0.63 per 100 py for MI, 1.56 vs 1.33 per 100 py for stroke or TIA, 6.07 vs 3.07 per 100 py for all-cause mortality, 4.00 vs 1.85 per 100 py for cardiovascular death, and 2.03 vs 1.22 per 100 py for non-cardiovascular death. After multivariable adjustment, we did not find conclusive evidence of increased rate of secondary outcomes in patients with PM compared to patients with no PM.

## Discussion

In this large cohort of unselected patients with AF, patients with a PM experienced nearly double the rates of MACE and HF hospitalizations compared to those without a PM. Over a 7-year FU, 27% of AF patients with a PM experienced MACE, and 22% were hospitalized for HF. This substantially increased risk was consistent across most of the secondary outcomes. However, after comprehensive adjusting for cardiovascular risk factors and comorbidities, the presence of a PM itself was not independently associated with these adverse outcome events, although a slight increase in risk cannot be excluded. These results suggest that AF patients with a PM are high-risk individuals, with the increased risk primarily driven by associated comorbidities. To the best of our knowledge, this is the first study to assess these associations in such a population.

In our cohort, approximately one out of eight patients with AF had a PM, with an annual implantation rate of 1%. In comparison, the Stockholm Cohort Study of Atrial Fibrillation with 2824 participants and a mean age of 74 (±12) years reported a 10% PM prevalence, similar to this study.^29^ Likewise, the RECORDAF-study (*n* = 5604; mean age of 66 ± 11.9 years) found an annual PM implantation rate of 2%.^30^ Considering that the prevalence of AF in Europe is projected to reach 16–18 million people by 2060, clinicians will increasingly face a substantial patient population with AF and implanted PM.^10–12^

Compared to AF patients without a PM, the incidence rates suggest that those with a PM had 1.8 times higher rates of MACE and nearly double the rates of HF hospitalization. Over 7 years of FU, about one out of four AF patient with a PM experienced a MACE and one out of five was hospitalized for HF. Thus, AF patients with a PM present a high-risk group in the AF population. Patients with a PM were older and had more comorbidities, including HF, prior stroke, and renal impairment. After adjusting for these covariables, the presence of a PM itself was not associated with these adverse outcomes, although a slight risk increase cannot be ruled out. As HF is the most common cause of death in patients with AF, accounting for 30%, and stroke accounting for 8% of total death,^31^ it is conceivable that these comorbidities drive adverse outcomes in PM patients. Besides comorbidities, frailty may also be a major determinant of adverse outcomes in PM patients. Frailty is highly prevalent in elderly AF patients, associated with conduction system disorders and an independent risk factor for death.^32–34^ The presence of a PM may therefore indicate a higher risk for adverse outcomes in patients with a high comorbidity burden and frailty. These patients may benefit from intensified FU and management, for example, in specialized AF clinic, following the ABC-integrated AF management pathway.^32,35,36^

Our current findings are limited to patients with traditional right ventricular pacing and might not apply to leadless pacing or conduction system pacing.^19,37^ Specifically, conduction system pacing preserves or restores physiological ventricular activation and might attenuate adverse outcomes compared to right ventricular pacing. In the large MELOS RELOADED study, left bundle branch area pacing was associated with improved survival compared to right ventricular pacing over a 4-year FU in more than 3000 patients with a left ventricular ejection fraction of >40% and >20% pacing burden.^38^ In the recent CSPACE randomized controlled trial, conduction system pacing was similarly superior with regard to a composite outcome of pacemaker-induced cardiomyopathy, CRT upgrade, HF hospitalization, and all-cause mortality compared to right ventricular septal pacing in 202 patients with AV block.^39^ These results suggest that conduction system pacing might also be beneficial in unselected AF patients with a relevant ventricular pacing burden.

### Study strength and limitations

The strengths of this study include the large and well-characterized cohort of patients with AF, minimal missing data, a long FU period, and centralized adjudication of outcome events. Several potential limitations must be considered when interpreting the results. There is risk of residual confounding or bias. While information on the type of cardiac device, implantation date, and complications was available, data on pacemaker settings, percentage of ventricular or atrial pacing, or AV node ablation was lacking, which might have influenced the association of PM with adverse outcomes.

### Conclusions

Patients with AF and a PM had substantially higher event rates for MACE and hospitalization for HF compared to AF patients without a PM. The presence of a PM itself was not independently associated with adverse outcomes. Our data suggest that AF patients with a PM represent a high-risk group within the AF population and may therefore benefit from more intensive FU and target treatment strategies to improve their clinical outcomes.

## Data Availability

The data underlying this article will be shared on reasonable request to the corresponding author.
